# Identification of significant genes with poor prognosis in ovarian cancer via bioinformatical analysis

**DOI:** 10.1186/s13048-019-0508-2

**Published:** 2019-04-22

**Authors:** Hao Feng, Zhong-Yi Gu, Qin Li, Qiong-Hua Liu, Xiao-Yu Yang, Jun-Jie Zhang

**Affiliations:** 1 Department of Gynecology and Obstetrics, Obstetrics and Gynecology Hospital of Fudan University, #128 Shenyang Road, Shanghai, 200090 China; 2 Department of Gynaecology and Obstetrics, Changhai Hospital, Navy Medical University, #168 Changhai Road, Shanghai, 200433 China; 3 Department of Gynaecology, Aoyang Hospital Affiliated to Jiangsu University, #279 Jingang Road, Zhangjiagang, 215600 Jiangsu China; 4 Department of Hepatic Surgery, Eastern Hepatobiliary Surgery Hospital, Navy Medical University, #225 Changhai Road, Shanghai, 200438 China

**Keywords:** Bioinformatical analysis, Microarray, Ovarian cancer, Differentially expressed gene

## Abstract

Ovarian cancer (OC) is the highest frequent malignant gynecologic tumor with very complicated pathogenesis. The purpose of the present academic work was to identify significant genes with poor outcome and their underlying mechanisms. Gene expression profiles of GSE36668, GSE14407 and GSE18520 were available from GEO database. There are 69 OC tissues and 26 normal tissues in the three profile datasets. Differentially expressed genes (DEGs) between OC tissues and normal ovarian (OV) tissues were picked out by GEO2R tool and Venn diagram software. Next, we made use of the Database for Annotation, Visualization and Integrated Discovery (DAVID) to analyze Kyoto Encyclopedia of Gene and Genome (KEGG) pathway and gene ontology (GO). Then protein-protein interaction (PPI) of these DEGs was visualized by Cytoscape with Search Tool for the Retrieval of Interacting Genes (STRING). There were total of 216 consistently expressed genes in the three datasets, including 110 up-regulated genes enriched in cell division, sister chromatid cohesion, mitotic nuclear division, regulation of cell cycle, protein localization to kinetochore, cell proliferation and Cell cycle, progesterone-mediated oocyte maturation and p53 signaling pathway, while 106 down-regulated genes enriched in palate development, blood coagulation, positive regulation of transcription from RNA polymerase II promoter, axonogenesis, receptor internalization, negative regulation of transcription from RNA polymerase II promoter and no significant signaling pathways. Of PPI network analyzed by Molecular Complex Detection (MCODE) plug-in, all 33 up-regulated genes were selected. Furthermore, for the analysis of overall survival among those genes, Kaplan–Meier analysis was implemented and 20 of 33 genes had a significantly worse prognosis. For validation in Gene Expression Profiling Interactive Analysis (GEPIA), 15 of 20 genes were discovered highly expressed in OC tissues compared to normal OV tissues. Furthermore, four genes (BUB1B, BUB1, TTK and CCNB1) were found to significantly enrich in the cell cycle pathway via re-analysis of DAVID. In conclusion, we have identified four significant up-regulated DEGs with poor prognosis in OC on the basis of integrated bioinformatical methods, which could be potential therapeutic targets for OC patients.

## Background

Ovarian cancer (OC) is the fifth cause of cancerous death among women all over the world [1]. Although some prognostic biomarkers have been exploited, the overall survival of OC remains weak due to its difficulty in early detection, distant metastasis and rapid dissemination [2, 3]. Therefore, more reliable prognostic biomarkers should be explored as a target for improving the treatment effect and better understanding the underlying mechanism.

Gene chip which was used for more than ten years can quickly detect differentially expressed genes and was proved to be a reliable technique [4] that could make many slice data be produced and stored in public databases. Therefore, a large number of valuable clues could be explored for new research on the base of these data. Furthermore, many bioinformatical studies on OC have been produced in recent years [5], which proved that the integrated bioinformatical methods could help us to further study and better exploring the underlying mechanisms.

In this study, first, we chosed GSE36668, GSE18520 and GSE14407 from Gene Expression Omnibus (GEO). Second, we applied for GEO2R online tool and Venn diagram software to obtain the commonly differentially expressed genes (DEGs) in the three datasets above. Third, the Database for Annotation, Visualization and Integrated Discovery (DAVID) was used to analyze these DEGs including molecular function (MF), cellular component (CC), biological process (BP) and Kyoto Encyclopedia of Gene and Genome (KEGG) pathways. Fourth, we established protein-protein interaction (PPI) network and then applied Cytotype MCODE (Molecular Complex Detection) for additional analysis of the DEGs which would identify some core genes. Moreover, these core DEGs were imported into the Kaplan Meier plotter online database for the significant prognostic information (*P* < 0.05). In addition, we furtherly validated the DEGs expression between OV cancer tissues and normal OV tissues via Gene Expression Profiling Interactive Analysis (GEPIA) (*P* < 0.05). Taken above, only 15 DEGs were qualified. Then, we re-analyzed these 15 DEGs for KEGG pathway enrichment. Finally, four DEGs (BUB1B, BUB1, TTK and CCNB1) were generated and significantly enriched in the cell cycle pathway especially in G2/M phase. In conclusion, the bioinformatic study of our study provides some additional useful biomarkers which could be an effective target for OC patients.

## Methods

### Microarray data information

NCBI-GEO is regarded as a free public database of microarray/gene profile and we obtained the gene expression profile of GSE36668, GSE18520 and GSE 14407 in ovarian cancer and normal ovarian tissues. Microarray data of GSE36668, GSE18520 and GSE14407 were all on account of GPL570 Platforms ([HG-U133_Plus_2] Affymetrix Human Genome U133 Plus 2.0 Array) which included 4 OC tissues and 4 normal OV tissues, 53 OC tissues and 10 normal OV tissues and 12 OC tissues and 12 normal OV tissues, respectively.

### Data processing of DEGs

DEGs between OC specimen and normal OV specimen were identified via GEO2R online tools [6] with |logFC| > 2 and adjust *P* value < 0.05. Then, the raw data in TXT format were checked in Venn software online to detect the commonly DEGs among the three datasets. The DEGs with log FC < 0 was considered as down-regulated genes, while the DEGs with log FC > 0 was considered as an up-regulated gene.

### Gene ontology and pathway enrichment analysis

Gene ontology analysis (GO) is a commonly used approach for defining genes and its RNA or protein product to identify unique biological properties of high-throughput transcriptome or genome data [7]. KEGG is a collection of databases dealing with genomes, diseases, biological pathways, drugs, and chemical materials [8]. DAVID which is an online bioinformatic tool is designed to identify a large number of genes or proteins function [9]. We could use DAVID to visualize the DEGs enrichment of BP, MF, CC and pathways (*P* < 0.05).

### PPI network and module analysis

PPI information can be evaluated by an online tool, STRING (Search Tool for the Retrieval of Interacting Genes) [10]. Then, the STRING app in Cytoscape [11] was applied to examine the potential correlation between these DEGs (maximum number of interactors = 0 and confidence score ≥ 0.4). In addition, the MCODE app in Cytoscape was used to check modules of the PPI network (degree cutoff = 2, max. Depth = 100, k-core = 2, and node score cutoff = 0.2).

### Survival analysis and RNA sequencing expression of core genes

Kaplan Meier-plotter are a commonly used website tool for assessing the effect of a great number of genes on survival based on EGA, TCGA database and GEO (Affymetrix microarrays only) [12]. The log rank *P* value and hazard ratio (HR) with 95% confidence intervals were computed and showed on the plot. To validate these DEGs, we applied the GEPIA website to analyze the data of RNA sequencing expression on the basis of thousands of samples from the GTEx projects and TCGA [13].

## Results

### Identifcation of DEGs in ovarian cancers

**Fig. 1:**
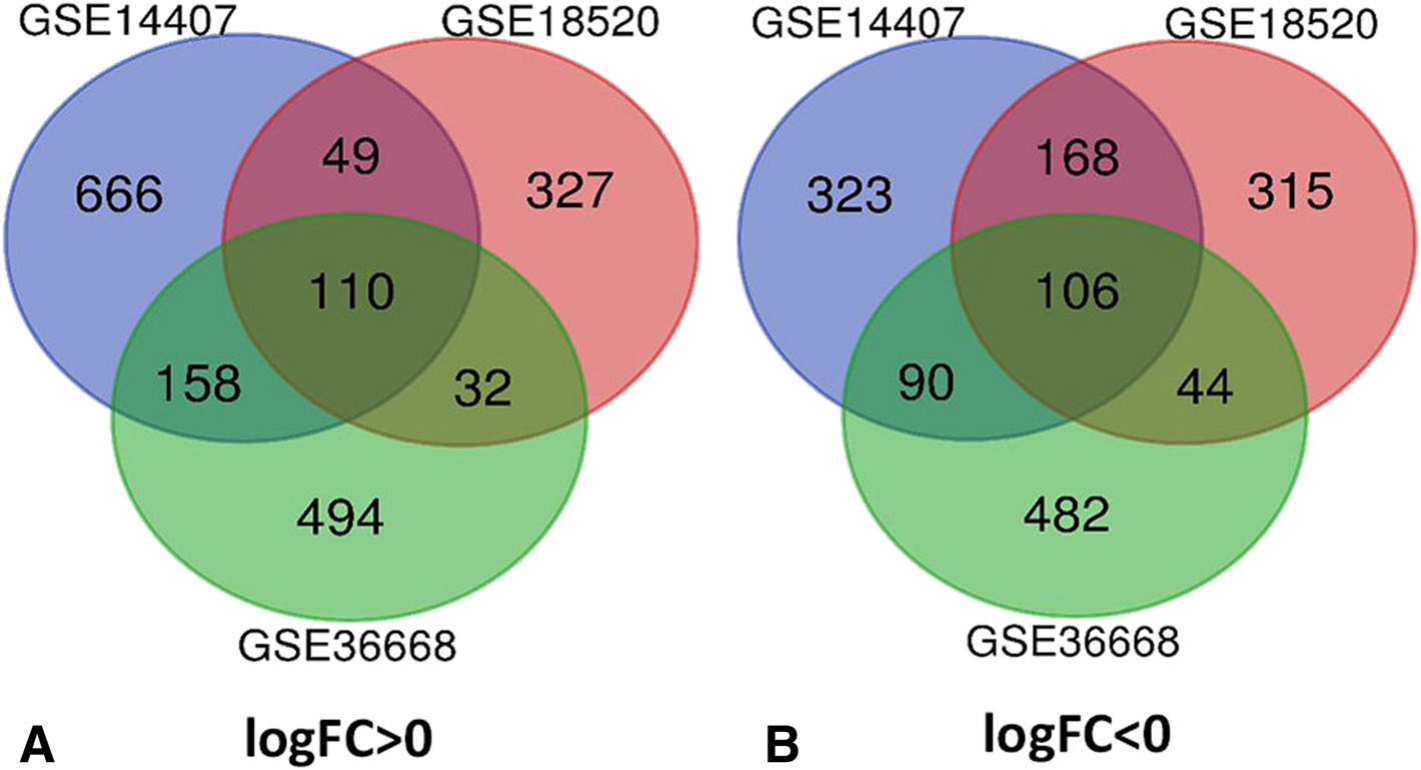
Authentication of 216 common DEGs in the three datasets (GSE36668, GSE18520 and GSE14407) through Venn diagrams software (available online: http://bioinformatics.psb.ugent.be/webtools/Venn/). Different color meant different datasets. **a** 110 DEGs were up-regulated in the three datasets (logFC> 0). **b** 106 DEGs were down-regulated in three datasets (logFC < 0)

There were 69 OC tissues and 26 normal OV tissues in our present study. Via GEO2R online tools, we extracted 1516, 1150 and 1670 DEGs from GSE36668, GSE18520 and GSE 14407, respectively. Then, we used Venn diagram software to identify the commonly DEGs in the three datasets. Results showed that a total of 216 commonly DEGs were detected, including 106 down-regulated genes (logFC< 0) and 110 up-regulated genes (logFC> 0) in the OC tissues (Table 1 & Fig. 1).

**Table 1:** All 216 commonly differentially expressed genes (DEGs) were detected from three profile datasets, including 106 down-regulated genes and 110 up-regulated genes in the OC tissues compared to normal OV tissues

DEGs	Genes Name
Up-regulated	C1orf106 MPZL2 EHF KLK6 MMP7 KLHL14 IGF2BP3 CCNB1 FOXQ1 PROM2 SUSD2 CLDN4 DEFB1 MEOX1 SMIM22 KLK8 FOXM1 CDK1 SORT1 MUC1 KIF11 ELF3 E2F1 FOLR1 MAL SULT1C2 CENPU STON2 GRHL2 KIF14 KCCAT333 AURKB MTHFD2 LOC101929219///LOC100505650///C1orf186 KIAA1217 KIF4A MCM10 CBS SOX17 EPHX4 CDH6 MELK CDC20 CXXC5 AIF1L DCDC2 INHBB BUB1 PRR11 TRIP13 CDCA5 SLC2A1 DUXAP10 EPCAM HMGA2 RGS1 ECT2 DEPDC1 MTFR2 LPAR3 UBE2C CCNB2 LOC100288637///ARHGAP11B CRABP2 CD24 LINC00673///LINC00511 PRSS2 LOC613266 TTC39A PRC1 PSAT1 LRP8 PTH2R RRM2 SLC35F6///CENPA TOP2A WDR72 S100A2 PAX8 KIF15 WFDC2 TFAP2A BUB1B TIMELESS NR2F6 MECOM RAD51AP1 ESCO2 LYNX1 ESRP1 DTL FAM83D HMMR C12orf56 GPM6B LOC101928554 CENPK LCN2 PRAME KIAA0101 HMGA1 TTK NCAPG CP SLC52A2 LINC01296///DUXAP10 NEK2 CENPF NUSAP1 ST6GALNAC1
Down-regulated	MUM1L1 NAP1L2 CYP2U1 VGLL3 GHR NEFH TMEM255A PPM1K TSPAN8 BAMBI MICU3 OC101930363///LOC101928349///LOC100507387///FAM153C///FAM153A///FAM153B LOC100507387///FAM153A///FAM153B BDH2 DPYD ANTXR2 HLF PRSS35 THBD PRRX1 LY75-CD302///CD302///LY75 ABCA8 WDR17 ZFPM2 OMD TCF21 PDGFD KLF2 SNCAIP NEGR1 NT5E RUNX1T1 TRPC1 SNCA PLEKHH2 GAS1RR MTUS1 GPM6A CPED1 MGARP LSAMP EFEMP1 B3GALT2 CHGB DIRAS3 PRKAR2B FAM13C KCNT2 TMEM150C ECM2 GIPC2 OGN SNX29P2 ARX TCEAL2 NAP1L3 SDPR TCEAL7 NBEA CXorf57 CSGALNACT1 CYS1 CNTN1 AKAP12 MEOX2 COL14A1 CALCRL ALDH1A1 SMPD3 TBX3 WNT2B ANKRD29 NR2F1-AS1 MCC CBLN4 CELF2 ITM2A GNG11 PGR OGFOD1 TFPI GPRASP1 PEG3 PCDH9 HAND2-AS1 RBMS3 FGF13 PRDM5 MAF PDE8B SIGLEC11 TLE4 DCN PEX5L BNC2 GATM RNF128 LHX9 AOX1 AKT3 OLFML1 RNASE4 GATA4 NXPH2 NDN LOC100506718///FLRT2

### DEGs gene ontology and KEGG pathway analysis in ovarian cancers

All 216 DEGs were analyzed by DAVID software and the results of GO analysis indicated that 1) for biological processes (BP), up-regulated DEGs were particularly enriched in regulation of cell cycle, cell division, mitotic nuclear division, protein localization to kinetochore, sister chromatid cohesion and cell proliferation, and down-regulated DEGs in blood coagulation, positive regulation of transcription from RNA polymerase II promoter, palate development, negative regulation of transcription from RNA polymerase II promoter, axonogenesis and receptor internalization; 2) for molecular function (MF), up-regulated DEGs were enriched in protein binding, ATP-dependent microtubule motor activity, protein kinase binding, plus-end-directed, microtubule binding, sequence-specific DNA binding and down-regulated DEGs in RNA polymerase II core promoter proximal region sequence-specific binding, RNA polymerase II transcription factor binding, RNA polymerase II core promoter proximal region sequence-specific binding and growth factor activity, transcriptional repressor activity, transcriptional activator activity; 3) for GO cell component (CC), up-regulated DEGs were significantly enriched in the nucleoplasm, midbody, spindle microtubule, spindle, cytosol and nucleus, and down-regulated DEGs in proteinaceous extracellular matrix anchored component of membrane, extracellular region and extracellular space (Table 2).

**Table 2:** Gene ontology analysis of differentially expressed genes in ovarian cancer

Expression	Category	Term	Count	%	*p*-Value	FDR
Up-regulated	GOTERM_BP_DIRECT	GO:0051301~cell division	16	15.24	9.45E-10	1.43E-06
	GOTERM_BP_DIRECT	GO:0007067~mitotic nuclear division	14	13.33	1.21E-09	1.84E-06
	GOTERM_BP_DIRECT	GO:0007062~sister chromatid cohesion	8	7.62	1.76E-06	0.002667
	GOTERM_BP_DIRECT	GO:0051726~regulation of cell cycle	8	7.62	6.07E-06	0.009214
	GOTERM_BP_DIRECT	GO:0034501~protein localization to kinetochore	4	3.81	1.98E-05	0.030061
	GOTERM_BP_DIRECT	GO:0008283~cell proliferation	11	10.48	3.81E-05	0.057753
	GOTERM_CC_DIRECT	GO:0005654~nucleoplasm	37	35.24	8.26E-08	9.94E-05
	GOTERM_CC_DIRECT	GO:0030496~midbody	9	8.57	3.99E-07	4.80E-04
	GOTERM_CC_DIRECT	GO:0005876~spindle microtubule	6	5.71	3.37E-06	0.004057
	GOTERM_CC_DIRECT	GO:0005819~spindle	8	7.62	3.49E-06	0.004203
	GOTERM_CC_DIRECT	GO:0005829~cytosol	35	33.33	4.42E-05	0.053183
	GOTERM_CC_DIRECT	GO:0005634~nucleus	48	45.71	5.52E-05	0.066432
	GOTERM_MF_DIRECT	GO:0005515~protein binding	72	68.57	5.60E-07	7.08E-04
	GOTERM_MF_DIRECT	GO:0043565~sequence-specific DNA binding	11	10.48	5.14E-04	0.647343
	GOTERM_MF_DIRECT	GO:0008017~microtubule binding	7	6.67	9.63E-04	1.210835
	GOTERM_MF_DIRECT	GO:0008574~ATP-dependent microt-ubule motor activity, plus-end-directed	3	2.86	0.003788	4.685832
	GOTERM_MF_DIRECT	GO:0019901~protein kinase binding	8	7.62	0.0044544	5.488301
Down-regulated	GOTERM_BP_DIRECT	GO:0060021~palate development	5	4.91	4.95E-04	0.721802
	GOTERM_BP_DIRECT	GO:0007596~blood coagulation	6	5.89	0.001956	2.823695
	GOTERM_BP_DIRECT	GO:0045944~positive regulation of transcription from RNA polymerase II promoter	12	11.76	0.00728	10.1353
	GOTERM_BP_DIRECT	GO:0000122~negative regulation of transcription from RNA polymerase II promoter	10	9.80	0.00773	10.72979
	GOTERM_BP_DIRECT	GO:0007409~axonogenesis	4	3.92	0.011841	15.98884
	GOTERM_BP_DIRECT	GO:0031623~receptor internalization	3	2.94	0.018262	23.62882
	GOTERM_CC_DIRECT	GO:0031225~anchored component of membrane	5	4.90	0.002893	3.225113
	GOTERM_CC_DIRECT	GO:0005576~extracellular region	17	16.67	0.008777	9.4952
	GOTERM_CC_DIRECT	GO:0005578~proteinaceous extracellular matrix	6	5.88	0.013131	13.89424
	GOTERM_CC_DIRECT	GO:0005615~extracellular space	14	13.72	0.022365	22.58432
	GOTERM_MF_DIRECT	GO:0043565~sequence-specific DNA binding	10	9.80	0.00117	1.454993
	GOTERM_MF_DIRECT	GO:0001078~transcriptional repressor activity, RNA polymerase II core promoter proximal region sequence-specific binding	4	3.92	0.018506	20.85873
	GOTERM_MF_DIRECT	GO:0001085~RNA polymerase II transcription factor binding	3	2.94	0.023394	25.65558
	GOTERM_MF_DIRECT	GO:0001077~transcriptional activator activity, RNA polymerase II core promoter proximal region sequence-specific binding	5	4.90	0.031296	32.84726
	GOTERM_MF_DIRECT	GO:0008083~growth factor activity	4	3.92	0.048426	46.29421

KEGG analysis results were shown in Table 3 which demonstrated that up-regulated DEGs were particularly enriched in p53 signaling pathway, cell cycle and progesterone-mediated oocyte maturation while down-regulated DEGs in no significant signaling pathways (*P* < 0.05).

**Table 3:** KEGG pathway analysis of differentially expressed genes in ovarian cancer

Pathway ID	Name	Count	%	*p*-Value	Genes
hsa04110	Cell cycle	8	7.62	7.31E-07	CCNB1, E2F1, CDK1, CCNB2, BUB1, TTK, BUB1B, CDC20
hsa04115	p53 signaling pathway	4	3.81	0.002934	CCNB1, CDK1, CCNB2, RRM2
hsa04914	Progesterone-mediated oocyte maturation	4	3.81	0.006123	CCNB1, CDK1, CCNB2, BUB1

### Protein–protein interaction network (PPI) and modular analysis

**Fig. 2:**
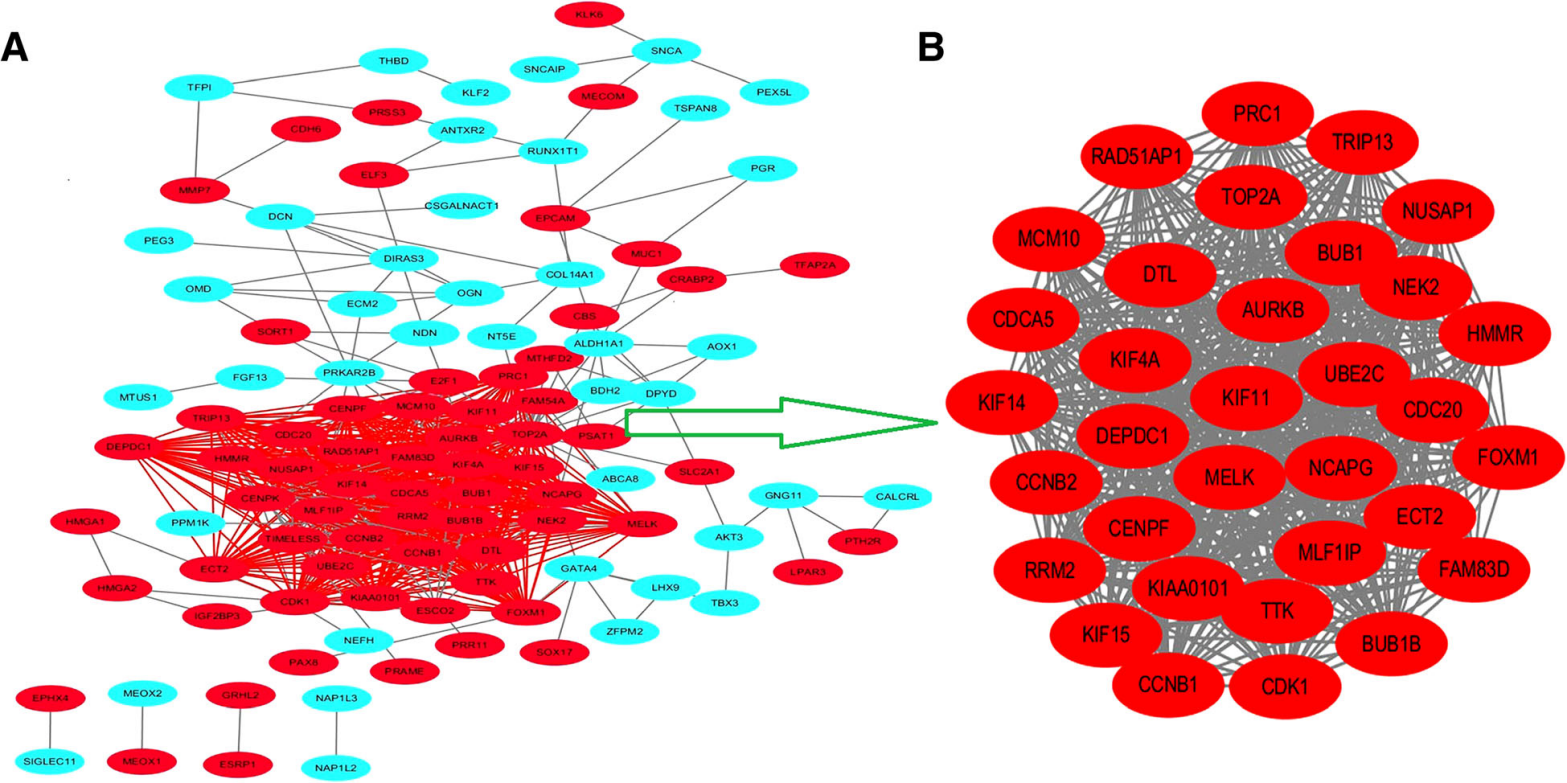
Common DEGs PPI network constructed by STRING online database and Module analysis. **a** There were a total of 107 DEGs in the DEGs PPI network complex. The nodes meant proteins; the edges meant the interaction of proteins; green circles meant down-regulated DEGs and red circles meant up-regulated DEGs. **b** Module analysis via Cytoscape software (degree cutoff = 2, node score cutoff = 0.2, k-core = 2, and max. Depth = 100)

A total of 107 DEGs were imported into the DEGs PPI network complex which included 107 nodes and 698 edges, including 60 down-regulated and 47 up-regulated genes (Fig. 2a). There were total 109 of the 216 DEGs which were not contained into the DEGs PPI network (Fig. 2a). Then we applied Cytotype MCODE for further analysis and results showed that 33 central nodes which were all up-regulated genes were identified among the 107 nodes (Fig. 2b).

### Analysis of core genes by the Kaplan Meier plotter and GEPIA

**Fig. 3:**
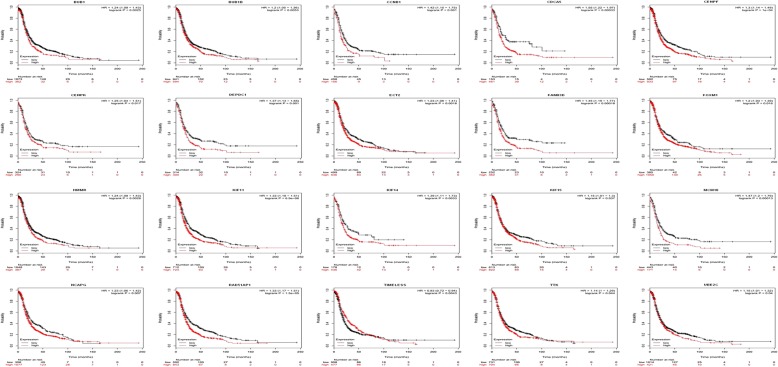
The prognostic information of the 33 core genes. Kaplan meier plotter online tools were used to identify the prognositc information of the 33 core genes and 20 of 33 genes had a significantly worse survival rate (*P* < 0.05)

**Fig. 4:**
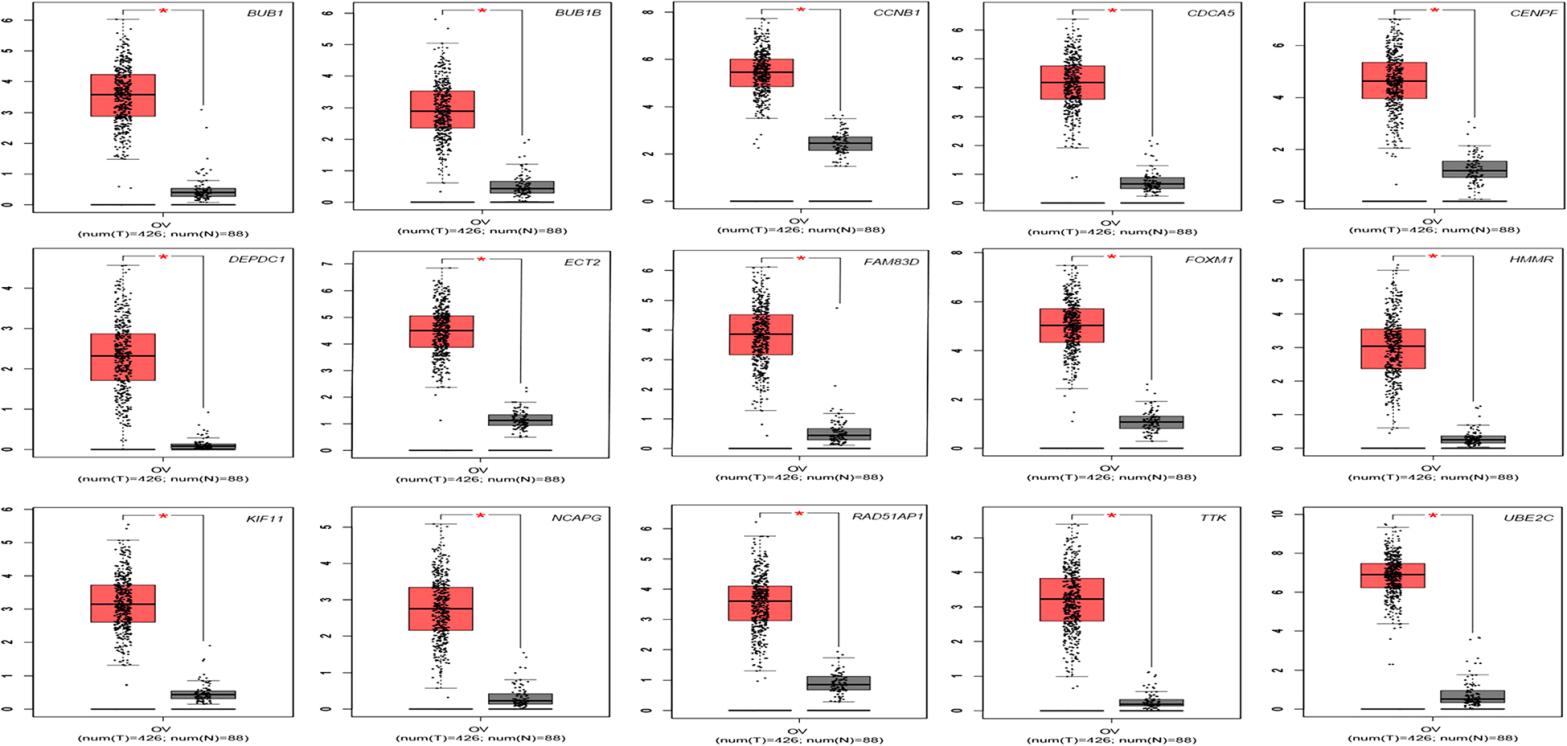
Significantly expressed 20 genes in OV cancer patients compared to healthy people. To further identify the genes’ expression level between OV cancer and normal people, 20 genes which were related with poor prognosis were analyzed by GEPIA website. 15 of 20 genes had significant expression level in OV cancer specimen compared to normal specimen (^*^P < 0.05). Red color means tumor tissues and grey color means normal tissues

Kaplan Meier plotter (http://kmplot.com/analysis) was utilized to identify 33 core genes survival data. It was found that 20 genes had a significantly worse survival while 13 had no significant (*P* < 0.05, Table 4 & Fig. 3). Then, GEPIA was used to dig up the 20 gene expression level between cancerous and normal people. Results reported that 15 of 20 genes reflected high expressed in OC samples contrasted to normal OV samples (*P* < 0.05, Table 5 & Fig. 4).

**Table 4:** The prognostic information of the 33 key candidate genes

Category	Genes
Genes with significantly worse survival (*P* < 0.05)	BUB1 BUB1B CCNB1 CDCA5 CENPF CENPK DEPDC1 ECT2 FAM83D FOXM1 HMMR KIF11 KIF14 KIF15 MCM10 NCAPG RAD51AP1 TIMELESS TTK UBE2C
Genes without significantly worse survival (*P* > 0.05)	AURKB CCNB2 CDC20 DTL E2F1 KIAA0101 KIF4A MELK NEK2 NUSAP1 PRC1 RRM2 TRIP13

**Table 5:** Vadidation of 20 genes via GEPIA

Category	Genes
Genes with high expressed in OC (*P* < 0.05)	BUB1 BUB1B CCNB1 CDCA5 CENPF DEPDC1 ECT2 FAM83D FOXM1 HMMR KIF11 NCAPG RAD51AP1 TTK UBE2C
Genes without high expressed in OC (*P* > 0.05)	CENPK KIF14 KIF15 MCM10 TIMELESS

### Re-analysis of 15 selected genes via KEGG pathway enrichment

**Fig. 5:**
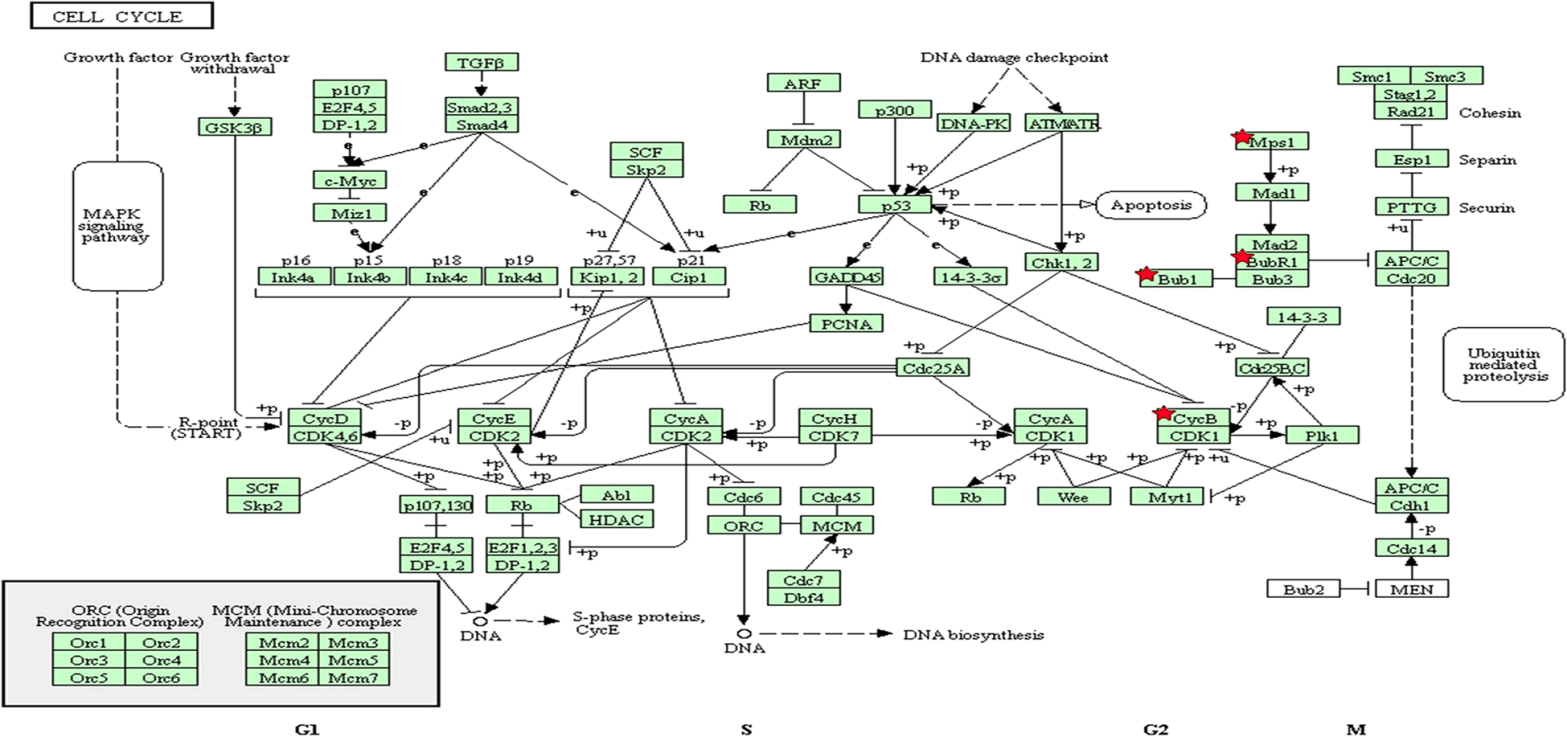
Re-analysis of 15 selected genes by KEGGpathway enrichment. Fifteen high expressed genes in OV cancer tissues with poor prognosis were re-analyzed by KEGG pathway enrichment. Four genes (BUB1B,BUB1,TTK and CCNB1) were significantly enriched in the cell cycle pathway especially in G2/M phase. Mps1 means TTK. BubR1 means BUB1B. CycB means CCNB1

To understand the possible pathway of these 15 selected DEGs, KEGG pathway enrichment was re-analyzed via DAVID (*P* < 0.05). Results showed that four genes (BUB1B, BUB1, TTK and CCNB1) markedly enriched in the cell cycle pathway (*P* = 1.1E-4, Table 6 & Fig. 5).

**Table 6:** Re-analysis of 15 selected genes via KEGG pathway enrichment

Pathway ID	Name	Count	%	*p*-Value	Genes
hsa04110	Cell cycle	4	26.7	1.1E-04	CCNB1 BUB1 TTK BUB1B
hsa04914	Progesterone-mediated oocyte maturation	2	13.3	7.3E-02	CCNB1 BUB1

## Discussion

To identify more useful prognostic biomarkers in OV cancer, this study used bioinformatical methods on the basis of three profile datasets (GSE36668, GSE18520 and GSE 14407). Sixty-nine ovarian cancer specimens and twenty-six normal specimens were enrolled in the present research. Via GEO2R and Venn software, we revealed a total of 216 commonly changed DEGs (|logFC| > 2 and adjust *P* value < 0.05) including 110 up-regulated (Log FC > 0) and 106 down-regulated DEGs (Log FC < 0). Then, Gene Ontology and Pathway Enrichment Analysis using DAVID methods showed that 1) for biological processes (BP), up-regulated DEGs were particularly enriched in regulation of cell cycle, cell division, mitotic nuclear division, protein localization to kinetochore, sister chromatid cohesion and cell proliferation, and down-regulated DEGs in blood coagulation, positive regulation of transcription from RNA polymerase II promoter, palate development, negative regulation of transcription from RNA polymerase II promoter, axonogenesis and receptor internalization; 2) for molecular function (MF), up-regulated DEGs were enriched in ATP-dependent microtubule motor activity, protein binding, plus-end-directed, microtubule binding, sequence-specific DNA binding, protein kinase binding and down-regulated DEGs in transcriptional repressor activity, RNA polymerase II core promoter proximal region sequence-specific binding and growth factor activity, RNA polymerase II core promoter proximal region sequence-specific binding, RNA polymerase II transcription factor binding, transcriptional activator activity; 3) for GO cell component (CC), up-regulated DEGs were significantly enriched in the nucleoplasm, midbody, spindle microtubule, spindle, cytosol and nucleus, and down-regulated DEGs in proteinaceous extracellular matrix anchored component of membrane, extracellular space and extracellular region. For pathway analysis, up-regulated DEGs were particularly enriched in p53 signaling pathway, cell cycle and progesterone-mediated oocyte maturation and while down-regulated DEGs in no noteworthy signaling pathways (*P* < 0.05). Next, DEGs PPI network complex of 108 nodes and 698 edges was constructed via the STRING online database and Cytoscape software. Then, 33 vital up-regulated genes were screened from the PPI network complex by Cytotype MCODE analysis. Furthermore, through Kaplan Meier plotter analysis, we found that 20 of 33 genes had a significantly worse survival. In validating these 20 genes, 15 genes reflected high expression in OC samples compared with normal samples by GEPIA analysis (*P* < 0.05). Finally, we re-analyzed 15 genes via DAVID for KEGG pathway enrichment and found that four genes (BUB1B, BUB1, TTK and CCNB1) enriched in cell cycle had a significance (*P* < 0.05) which could be considered as new effective targets to improve the prognosis of OC patients.

Mitotic checkpoint serine/threonine kinase B (BUB1B), which is seen as a mammalian homolog of yeast Mad3, but they are significantly different because BUB1B has a kinase domain which is not found in Mad3 [14]. In 2004, Kops GJ, et al. reported that apoptotic cell death and massive chromosome loss could occur due to the inhibition of BUB1B kinase activity and reduction of the BUB1B level in human cancer cells [15]. BUB1B has been demonstrated to enhance tumor proliferation and is associated with worse survival rate in several types of cancer, including prostate cancer, breast, gastric and colorectal [16–19]. Another study proved that knockdown of BUB1B resulted in inhibition of tumor growth in vivo, including the regression of established tumors via postmitotic endoreduplication checkpoint [20] which is the replication of the genome during the cell cycle without the subsequent completion of mitosis and/or cytokinesis [21].

BUB1 which is a serine/threonine kinase and encoded by the BUB1 gene, binds centromeres during mitosis. It has been noted that over-expressed BUB1 is related to several cancers and their worse clinical prognosis. Wang et al. [22] presented that high expression of BUB1 was associated with poor disease-free survival of 203 patients with breast cancer. In addition, Zhao et al. [23] indicated that higher positive percentage of BUB1 protein meant a more advanced stage and a higher differentiation degree of endometrial carcinoma. Furthermore, Pinto et al. [24] demonstrated that over-expression of BUB1 was found to be substantially related to Furhman grade of the tumors and with the number of genomic copy number changes. By isolating daughter cells from mother cells, BUB1 also were vitally responsible for the accurate assignment of chromosomes without establishing the mitotic spindle checkpoint and aligning chromosomes [25, 26].

Monopolar spindle1 (Mps1, also known as TTK), is a bispecific protein kinase that phosphorylates serines/threonines and tyrosines [27]. Mps1 is a core segment of the SAC (spindle assembly checkpoint) and is a key monitoring mechanism to ensure healthy cell proliferation and precise division [28, 29]. In addition to mitotic SAC regulation, Mps1 play roles in other processes, including DNA damage response, centrosome duplication and organ development [30]. Moreover, high expression of Mps1 was easily found in several human malignancies, such as thyroid carcinoma, glioblastoma and breast cancer [31–34].

CCNB1, G2/Mitotic-specific cyclin B1, is a monitoring protein in mitosis and expressed primarily in G2/M phase which is critical for controlling the cell cycle at the G2/M (mitosis) transition. Recently, increasing evidence demonstrated that CCNB1 was over-expressed in considerable cancers with poor prognosis, including gastric cancer [35], esophageal squamous cell carcinoma [36], non-small cell lung cancer [37] and astrocytomas [38]. Furthermore, it was also pointed out that down-regulation of CCNB1 of mRNA levels and protein could reduce cell proliferation [39]. In 2017, Zhao P, et al. reported that up-regulation of CCNB1 could be an index for pituitary adenomas invasiveness and played a part in the pathology of pituitary adenomas with other monitoring molecules in the cell cycle [40].

Numerous studies have proved that these four genes were related to various types of cancer’s progression, however, very few studies have been reported about these four genes in OV cancer after we searched these four genes in Pubmed website. Therefore, the data in our study could provide useful information and direction for future study in OV cancer.

## Conclusions

Taken above, our bioinformatics analysis study identified four DEGs (BUB1B, BUB1, TTK and CCNB1) between OC tissues and normal OV tissues on the base of three different microarray datasets. Results showed that these four genes could play key roles in the progression of OC. However, these predictions should be verified by a series of experiments in the future. Anyway, these data may provide some useful information and direction into the potential bio-markers and biological mechanisms of OC.

## Abbreviations

BP: Biological process
BUB1B: Mitotic checkpoint serine/threonine kinase B
CC: Cellular component
CCNB1: G2/Mitotic-specific cyclin B1
DAVID: Database for Annotation, Visualization and Integrated Discovery
DEGs: Differentially expressed genes
GEO: Gene Expression Omnibus
GEPIA: Gene Expression Profiling Interactive Analysis
GO: Gene ontology
KEGG: Kyoto Encyclopedia of Gene and Genome
MCODE: Molecular Complex Detection
MF: Molecular function
OC: Ovarian cancer
OV: Ovarian
PPI: protein-protein interaction
STRING: Search Tool for the Retrieval of Interacting Genes
